# Supplementary material for: Hybrid Models and Biological Model Reduction with PyDSTool
Source: PLoS Comput Biol. 2012 Aug 9;8(8):e1002628. doi: 10.1371/journal.pcbi.1002628 (PMC3415397; doi:10.1371/journal.pcbi.1002628)
Supplement: Text S4 — Complete source code for the PyDSTool package (version 0.88.120504). Includes API documentation and help files linking to web pages. This file is identical to the current public release on Sourceforge.net. (ZIP) [file pcbi.1002628.s004.zip › PyDSTool/html/PyDSTool.Toolbox.InputProfile-pysrc.html]

xml version="1.0" encoding="ascii"?


PyDSTool.Toolbox.InputProfile


| Home | Trees | Indices | Help | PyDSTool |
| --- | --- | --- | --- | --- |

Package PyDSTool ::
Package Toolbox ::
Module InputProfile

[hide private]

[frames] | no frames]

# Source Code for Module PyDSTool.Toolbox.InputProfile

```
  1  from PyDSTool import * 
  2   


3 -def makeSpikeProfile(rawT, rawX, baseline=0.0, upshift=0.0, peak='max', endTime=1000000):


4       
  5      profile = {} 
  6      assert (len(rawT) > 2), 'profiles must have length > 2' 
  7      assert (len(rawT) == len(rawX)), 'dep. and indep. variable profiles must have same length.' 
  8       
  9      if peak == 'max': 
 10          n = rawX.index(max(rawX)) 
 11          m = rawX.index(min(rawX)) 
 12      else: 
 13          n = rawX.index(min(rawX)) 
 14          m = rawX.index(max(rawX)) 
 15           
 16      # Determine starting time from secant to peak 
 17      if rawX[0] == baseline: 
 18          startT = [rawT[0] - (rawT[1] - rawT[0])] 
 19      else: 
 20          startT = [(baseline - rawX[0] - upshift) * (rawT[n] - rawT[0])/(rawX[n] - rawX[0]) 
 21                    + rawT[0]] 
 22      if rawX[-1] == baseline: 
 23          endT = [rawT[-1] + rawT[-1] - rawT[-2]] 
 24      else: 
 25          endT =  [(baseline - rawX[n] - upshift) * (rawT[-1] - rawT[n])/(rawX[-1] - rawX[n]) 
 26                   + rawT[n]] 
 27       
 28      profile['t'] = array( startT + rawT + endT ) - startT[0] 
 29      if upshift != 0: 
 30          shiftX = [x + upshift for x in rawX] 
 31      else: 
 32          shiftX = rawX 
 33      profile['x'] = array( [baseline] + shiftX + [baseline] ) 
 34       
 35      profile['peakT'] = profile['t'][n + 1] 
 36      profile['peakX'] = profile['x'][n + 1] 
 37      profile['peakIdx'] = n+1 
 38       
 39      profile['perturbWhole'] = profile['t'][-1] - profile['t'][0] 
 40      profile['perturbMain'] = profile['t'][-2] - profile['t'][1] 
 41       
 42      profile['spikeHeight_base'] = abs(rawX[n] - baseline) 
 43      profile['spikeHeight_spike'] = abs(rawX[n] - rawX[m]) 
 44   
 45      profile['halfHeight_base'] = 0.5 * profile['spikeHeight_base'] + baseline 
 46      profile['halfHeight_spike'] = 0.5 * profile['spikeHeight_spike'] + baseline 
 47       
 48      profile['baseWidth'] = abs(rawT[-1] - rawT[0]) 
 49      profile['baseline'] = baseline 
 50       
 51      # Use interpolation table to determine spike half-width 
 52      upsign = 1 
 53      downsign = 1 
 54   
 55      if peak == 'max': 
 56          downsign = -1     
 57      else: 
 58          upsign = -1 
 59           
 60      upT = profile['t'][0:n+2] 
 61      downT = profile['t'][n+1:-1] 
 62       
 63      upX = upsign*profile['x'][0:n+2] 
 64      upX = upX.tolist() 
 65      downX = downsign*profile['x'][n+1:-1] 
 66      downX = downX.tolist() 
 67     
 68      uTable = InterpolateTable({'tdata': upX, 'ics': makeDataDict(['up'], [upT]), 'name': 'up'}) 
 69      dTable = InterpolateTable({'tdata': downX, 'ics': makeDataDict(['down'], [downT]), 'name': 'down'}) 
 70      uTraj = uTable.compute('up') 
 71      dTraj = dTable.compute('down') 
 72       
 73      profile['halfWidth_up'] = (uTraj(upsign*profile['halfHeight_base']), uTraj(upsign*profile['halfHeight_spike'])) 
 74      profile['halfWidth_down'] = (dTraj(downsign*profile['halfHeight_base']), dTraj(downsign*profile['halfHeight_spike'])) 
 75      profile['halfWidth_base'] = abs(profile['halfWidth_up'][0] - profile['halfWidth_down'][0]) 
 76      profile['halfWidth_spike'] = abs(profile['halfWidth_up'][1] - profile['halfWidth_down'][1]) 
 77       
 78      profile['eSynRev'] = 0.88317057588863412 * (profile['peakX'] - profile['baseline']) + profile['baseline'] 
 79      profile['iSynRev'] = profile['baseline'] 
 80      profile['halfAct'] = 0.54989866045896085 * (profile['peakX'] - profile['baseline']) + profile['baseline'] 
 81       
 82      if endTime is not None and endTime > profile['t'][-1]: 
 83          profile['t'] = array( profile['t'].tolist() + [endTime] ) 
 84          profile['x'] = array( profile['x'].tolist() + [profile['x'][-1]] ) 
 85       
 86      return profile

 87   


88 -def makeInputTable(profile, name):


89       
 90      xData = makeDataDict([name], [profile['x']]) 
 91      tData = profile['t'] 
 92      iTable = InterpolateTable({'tdata': tData, 'ics': xData, 'name': name}) 
 93       
 94      iTableTraj = iTable.compute(name) 
 95   
 96      return iTableTraj

 97   
 98   


99 -def plotProfile(profile, figure='new'):


100   
101      if figure == 'new': 
102          pylab.figure() 
103           
104      plot(profile['t'], profile['x'], 'b-') 
105      plot(profile['halfWidth_up'][0], profile['halfHeight_base'], 'b^') 
106      plot(profile['halfWidth_up'][1], profile['halfHeight_spike'], 'r^') 
107      plot(profile['halfWidth_down'][0], profile['halfHeight_base'], 'bo') 
108      plot(profile['halfWidth_down'][1], profile['halfHeight_spike'], 'ro') 
109   
110      plot(profile['peakT'], profile['peakX'], 'go')

111
```

  


| Home | Trees | Indices | Help | PyDSTool |
| --- | --- | --- | --- | --- |

|  |  |
| --- | --- |
| Generated by Epydoc 3.0.1 on Fri May 4 15:24:20 2012 | http://epydoc.sourceforge.net |
